# Fungus-larva relation in the formation of *Cordyceps sinensis* as revealed by stable carbon isotope analysis

**DOI:** 10.1038/s41598-017-08198-1

**Published:** 2017-08-10

**Authors:** Lian-Xian Guo, Yue-Hui Hong, Qian-Zhi Zhou, Qing Zhu, Xiao-Ming Xu, Jiang-Hai Wang

**Affiliations:** 1 Dongguan Key Laboratory of Environmental Medicine, School of Public Health, Guangdong Medical University, Dongguan, Guangdong, 523808 People’s Republic of China; 2 Guangdong Provincial Key Laboratory of Marine Resources and Coastal Engineering/South China Sea Bioresource Exploitation and Utilization Collaborative Innovation Center, School of Marine Sciences, Sun Yat-Sen University, Guangzhou, 510006 People’s Republic of China

## Abstract

For more than one thousand years, *Cordyceps sinensis* has been revered as a unique halidom in the Qinghai-Tibetan Plateau for its mysterious life history and predominant medicinal values. This mysterious fungus-larva symbiote also attracted the over-exploitation, while several problems on the initial colonization of *Ophiocordyceps sinensis* in the host larva have constrained artificial cultivation. In this work, stable carbon isotope analysis was employed to analyse the subsamples of *C*. *sinensis* from 5 representative habitats. The results demonstrated that these samples possessed similar δ^13^C profiles, i.e., a steady ascending trend from the top to the bottom of stroma, occurrence of the δ^13^C maximum at the head, a slight decrease from the head to the end of thorax, a sharply descent trend from the end of thorax to the forepart of abdomen, and maintenance of lower δ^13^C values in the rest parts of abdomen. Based on the data, we consider that the site near the head of the host larva may be the initial target attacked by *O*. *sinensis*, and the fungus growth is closely related to the digestive tract of its host larva. The growth stages of *O*. *sinensis* are accordingly speculated as the symptom-free, symptom-appearing, and stroma-germinating stages.

## Introduction

The mystery that a worm in winter will transform into one herb in summer has been recorded in Chinese traditional medicines for more than one thousand years. The worm is widely known as Chinese caterpillar fungus. This strange life form, popularly named as winter-worm-summer-grass (Dong Chong Xia Cao in Chinese), is a fungus-larva symbiote, and the “grass” is the fruiting body of *Cordyceps sinensis*. [Note: The Latin name *Cordyceps sinensis* (Berk.) Sacc. is used for both the fungus and the wild fungus-caterpillar complex product indiscriminately. The fungus has been re-named as *Ophiocordyceps sinensis* (Berk.)^1^; however, the Latin name for the wild product has remained unchanged. In this paper, we use *O*. *sinensis* to refer to the fungus/fungi, and *C*. *sinensis* to refer to the fungus-caterpillar complex.] *C*. *sinensis* undergoes a long-term and complicated life history^2^. In summer, the spores erupt from *O*. *sinensis* randomly and scatter in top soils. They develop into infective conidia, which gradually infiltrate into deeper soils mainly due to rainfall. The host^3^, a healthy *Thitarodes* (Lepidoptera: Hepialidae) larva, will overwinter in the alpine grass lands in the Qinghai-Tibetan Plateau and its adjacent high-altitude areas^4,5^, go deep into the roots of *Polygonum* knotweed, *Kobresia* sedges or *Astragalus* milk-vetch, and safely nestle in the roots of its favorite foods^6^. It may be infected by the fungus in the soils through the mouth or the skin. Then, the larva becomes a fungal host that is less excited, and the moribund larva moves sluggishly. Simultaneously, its skin colour gradually changes from brown to milky white. As a last rite of their union, the fungus directs its host larva to crawl into a position ideal for fungal spore dispersal^2,7^. The fungus will completely gut the interior of the larva, replacing its contents with thread-like hyphae. The larva progressively becomes stiff and is coated with mycelia. Although the remaining exoskeleton of the insect supports the illusion of a continued larval existence, by then it functions solely as a fungal food cache, ready to be completely raided when warmer temperatures allow the fungus to burst forth as a fruiting body right out of its fontanel (frontal region of the head capsule). This grass-like fruiting body will then serve as the means to disperse its millions of spores, thus initiating the next hostile take-over^2^.

*C*. *sinensis* has been used in China for more than 2,000 years as a rare health food and a traditional medicinal herb to promote health and treat diverse chronic diseases^8^. It exhibits evident beneficial effects on renal and hepatic functions and immunomodulation-related anti-tumor activities. Modern pharmacological studies have shown that *C*. *sinensis* possesses many chemical constituents with specific pharmacological activities, which have recently attracted much attention^9,10^. The pronounced medicinal function has resulted in a large demand for wild *C*. *sinensis*. However, its population is extremely limited due to its complicated life cycle, obligate parasitism and ecogeographical preferences^4,11^. Furthermore, the excessive excavation, human destruction of its habitats, and the upward movement of snow line due to global warming have further aggravated the yield decrease of *C*. *sinensis* in the latest twenty years^12^. The retail price of wild *C*. *sinensis* products has accordingly risen quickly. To alleviate the imbalance between its supply and demand, many investigators have focused on the large-scale artificial cultivation of *C*. *sinensis*.

However, the large-scale artificial cultivation of *C*. *sinensis* has not succeeded because several crucial problems on the developmental mechanism of *O*. *sinensis* colonizing in the host larva should be further clarified^13^, e.g., (i) where is the initially colonized position of *O*. *sinensis* in the host larva? (ii) what is the pathway to infect the host larva by *O*. *sinensis*? Some investigators considered that the spores of *O*. *sinensis* in soils initially attacked the newly-molted neck skin of its host larva. In its ecdysis stage, the infectious spores of *O*. *sinensis* might adhere to the injured skin or spiracle damage of the newly-molted larva. Then, the germinated spores developed into germ tubes and protruded through the skin or spiracle, and ultimately infected the host larva^14^. However, the other scholars proposed that the host larva was infected by the infectious spores of *O*. *sinensis*, which adhered to the tender plant roots as its foods^15^. Till now, there are two disputable hypotheses on the site of the initial colonies of *O*. *sinensis* in one larva, i.e., body surface infection and digestive system infection, and no hard experiment evidence is available to discriminate them. In addition, the infection pathway of the host larva by *O*. *sinensis* and the development of *C*. *sinensis* should further be expounded. To settle the above problems, the formers have done the field observations on wild host larvae, showing that they usually inhabit the upper soils at the depths of 15–35 cm or move upwards near the soil surface even if being infected by *O*. *sinensis* in late autumn, till after pupating^11^. Many artificial infection experiments of the larvae fed indoor have also been performed, revealing the changes in their morphology, pathology and behaviors from the infection to the formation of stiff worms as well as the hyphal growth in the hemocoelom^15,16^. Recently, some experts investigated the growth and reproduction of *O*. *sinensis in vitro*^17–19^, the genetic information of *C*. *sinensis* or other related entomopathogens^20,21^, and the transcriptome of larvae before and after being infected by *O*. *sinensis*^22^. However, these studies still failed to settle the above-mentioned problems, due to the complicated life cycle, strict growth environment of the host larva^23^, and the limitation of conventional research techniques.

**Figure 1:**
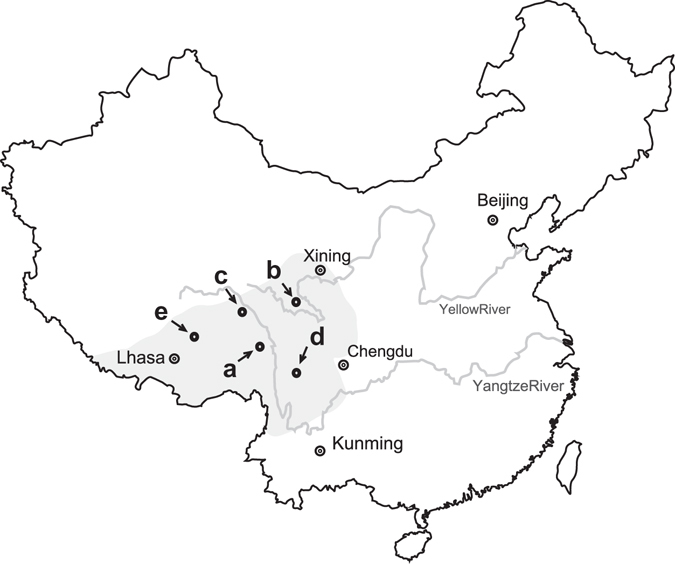
Schematic map illustrating the sampling sites in the Qinghai-Tibetan Plateau and its adjacent high-altitude areas. (**a**), Changdu; (**b**), Guoluo; (**c**), Haxiu; (**d**), Litang; and (**e**), Naqu. The map was modified from Guo, L. X. *et al*.^5^ (Copyright © 2011 Elsevier Masson SAS. All rights reserved.) using CorelDraw X4 (Corel Corporation, Ontario, Canada).

**Figure 2:**
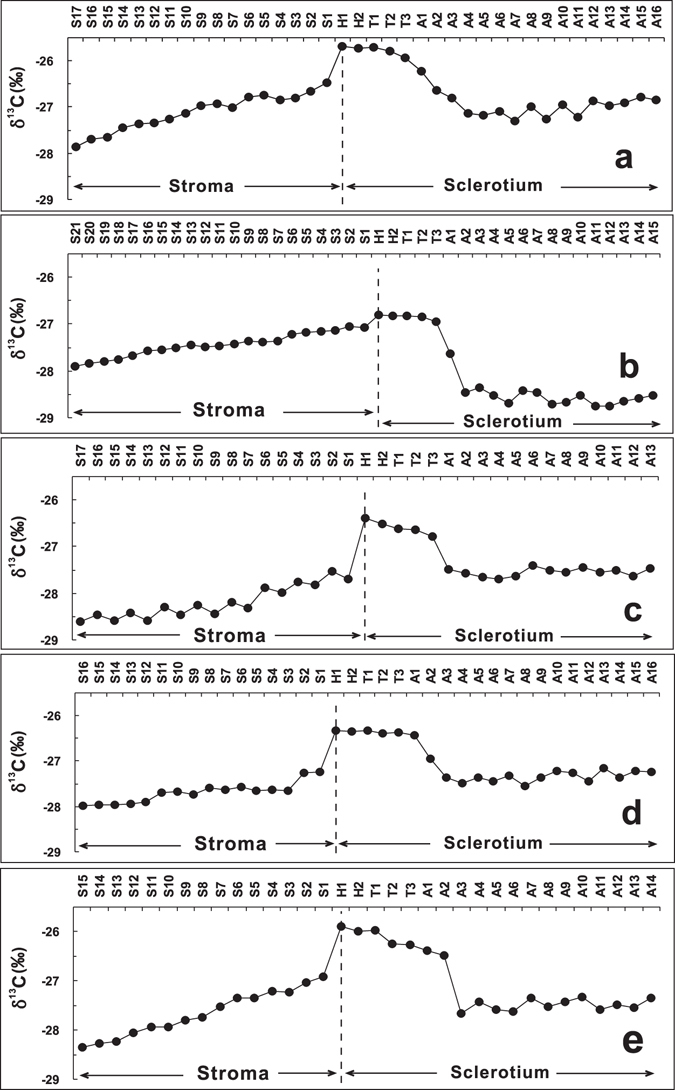
Variations of the δ^13^C values in the subsamples of *C*. *sinensis*. Samples (**a**,**b**,**c**,**d** and **e**) were produced from Changdu, Guoluo, Haxiu, Litang and Naqu, respectively. Each sample was sectioned into approximately 40 pieces from the stroma top to the sclerotium end. These sample sections were divided into four subsample groups according to their relative positions: stroma (S1 to S*i*), head (H1 to H2), thorax (T1 to T3), and abdomen (A1 to A*i*).

**Figure 3:**
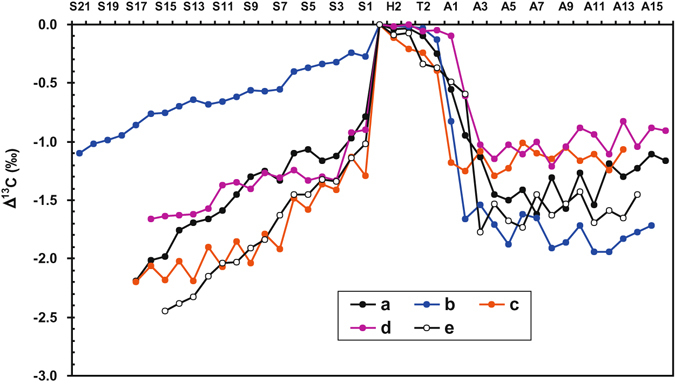
Variations of the Δ^13^C values between the δ^13^C values of the head section (H1) and others in *C*. *sinensis*. Samples (**a**,**b**,**c**,**d** and **e**) were produced from Changdu, Guoluo, Haxiu, Litang and Naqu, respectively. Each sample was sectioned into about 40 pieces from the stroma top to the sclerotium end. These sample sections contain four subsample groups according to their relative positions: stroma (S1 to S*i*), head (H1 to H2), thorax (T1 to T3), and abdomen (A1 to A*i*).

The difficulties in artificial cultivation, combined with the specific life history, the unusual distribution, and the prominent medicinal function, have made *C*. *sinensis* sacred and mysterious in local popular belief. In this paper, stable carbon isotope analysis is innovatively applied in revealing the mysterious veil. It is a powerful technique for tracing the biological metabolism and development. Besides, it has a remarkable advantage that it may provide the diachronic diet information of heterotrophic organisms and further study the exchange of their materials and energy with dispensable calibration^24–30^. Adopting a stable carbon isotope technique, our group reported the diet of the host larvae in the Sejila Mountain (Tibet), indicating that the humic substances in soils might be their potential foods in addition to the tender plant roots^6^. Till now, this technique has not been employed to study the infection and development of entomogenous fungi. For *C*. *sinensis*, the specific subterraneous living environments and complicated life histories of both *O*. *sinensis*^2^ and its host larva^11^ result in the inconvenient observations on its growth and development only on the basis of the existing techniques. In this paper, five samples of *C*. *sinensis* from its representative habitats in the Qinghai-Tibetan Plateau and its contiguous high-altitude districts (Fig. 1) were firstly dissected into approximately 40 subsamples for systematically measuring their stable carbon isotope ratios, and then the position of initial colonies of *O*. *sinensis* in the host larva and related processes were discussed on the basis of the profiles of the newly-obtained δ^13^C values (Fig. 2) and the differences of the δ^13^C values (Δ^13^C) between the head and other parts of *C*. *sinensis* (Fig. 3). Our result may provide a novel clue for revealing the developmental mechanism of *O*. *sinensis* and further contribute to achieving the large-scale artificial cultivation of *C*. *sinensis*.

## Results

### Stable carbon isotope composition of *C*. *sinensis*

The stable carbon isotope ratios for five representative samples of *C*. *sinensis* from different habitats in the Qinghai-Tibetan Plateau are presented in Supplementary Table S1, and their variations are illustrated in Fig. 2. It can been seen from Fig. 2 that the δ^13^C values of all samples of *C*. *sinensis* show a similar variation trend from the top of the stroma to the end of the caterpillar-shaped sclerotium (Spearman’s rank correlation analysis, *p* < 0.05). The δ^13^C values of the stroma show a stable increasing trend from the top to the bottom, and the δ^13^C values of the caterpillar-shaped sclerotium subsamples display a stepped downtrend from the head to the thorax, and to the abdomen end. The detailed variations are described as follows.

S*i* to H1: The δ^13^C values of the stroma show a stable increasing trend from the top to the bottom and range from −27.87‰ to −26.47‰ with a mean of −27.11‰ for sample a, from −27.91‰ to −27.08‰ with a mean of −27.44‰ for sample b, from −28.60‰ to −27.69‰ with a mean of −28.19‰ for sample c, from −27.99‰ to −27.23‰ with a mean of −27.20‰ for sample d, and from −28.35‰ to −26.92‰ with a mean of −27.67‰ for sample e, respectively. The δ^13^C values jump sharply from S1 to H1, and the change intervals vary among different samples of *C*. *sinensis*.

H1 to T3: The δ^13^C values of head and thorax always maintain in a higher step. The δ^13^C values of head (H1 and H2) are the highest and are at the intervals of −25.72‰ to −25.68‰ with an average of −25.70‰ for sample a, −26.83‰ to −26.81‰ with an average of −26.82‰ for sample b, −26.51‰ to −26.40‰ with an average of −26.46‰ for sample c, −26.35‰ to −26.33‰ with an average of −26.34‰ for sample d, and −25.99‰ to −25.90‰ with an average of −25.95‰ for sample e, respectively. The δ^13^C values of thorax (T1 to T3) exhibit a stable variation (samples b and d) or a slightly decreasing trend (samples a, c and e). The δ^13^C values of thorax are distributed between −25.71‰ and −25.93‰ with an average of −25.81‰ for sample a, −26.83‰ and −26.94‰ with an average of −26.87‰ for sample b, −26.61‰ and −26.79‰ with an average of −26.68‰ for sample c, −26.33‰ and −26.39‰ with an average of −26.37‰ for sample d, and −25.97‰ and −26.27‰ with an average of −26.16‰ for sample e, respectively.

T3 to A3: The δ^13^C values decrease dramatically from the end of thorax to the start of the third abdominal segment (T3 to A3), and their variations are at the intervals of −25.93‰ to −26.81‰ for sample a, −26.94‰ to −28.35‰ for sample b, −26.79‰ to −27.48‰ for sample c, −26.38‰ to −27.36‰ for sample d, and −26.27‰ to −27.67‰ for sample e, respectively.

A3 to A*i*: The δ^13^C values from A3 to A*i* stay in a steady lower step and are at the ranges of −26.79‰ to −27.30‰ with a mean of −27.02‰ for sample a, −28.35‰ to −28.75‰ with a mean of −28.59‰ for sample b, −27.41‰ to −27.69‰ with a mean of −27.54‰ for sample c, −27.16‰ to −27.54‰ with a mean of −27.34‰ for sample d, and −27.33‰ to −27.67‰ with a mean of −27.49‰ for sample e, respectively.

### Difference of the δ^13^C values between the head and other parts of *C*. *sinensis*

The difference of the δ^13^C values between head and other parts (Δ^13^C) may be employed to further reveal the spatial distribution of the δ^13^C values within *C*. *sinensis*. It can be seen from Table S1 and Fig. 3 that the Δ^13^C values of head fluctuate at a narrow interval of −0.02‰ to −0.11‰, while those of thorax vary from −0.02‰ to −0.39‰ with a detectable difference of 0.10−0.52‰, and those within abdomen vary from −0.10‰ to −1.94‰ with a detectable difference of 0.34−1.07‰. The Δ^13^C values between the head and stroma sections of *C*. *sinensis* vary from −0.27‰ to −2.45‰ with a detectable difference of 0.76−1.43‰, and there is a good negative linear relation between the Δ^13^C value and position (*i*) in each stroma of all samples (Pearson’s *r*² = 0.951, 0.970, 0.841, 0.811 and 0.987, respectively; *p* < 0.05).

## Discussion

The large-scale artificial cultivation of *C*. *sinensis* has not succeeded up to date, because several crucial problems on the developmental mechanism of *O*. *sinensis* colonizing in the host larva should be further clarified^13^. The formers have dissected numerous infected larvae in different infection stages for the macroscopic and microscopic studies on the fungus development^31^ (Table 1). However, previous studies cannot provide the detailed experimental data of mycelia development at the early infection stage and in the subsections of the infected host, because none of the studied larvae can survive from early to terminal infection stages. Obviously, stable carbon isotope analysis is suitable to perform a retrospective study^26^ on the fungus-larva relation in the formation of *C*. *sinensis*, just as shown in this paper. Therefore, the mechanisms of fungal development were put forward on the basis of combination of the newly obtained δ^13^C values and previous observations (Table 1).

**Table 1:** Comparison between stable carbon isotope analysis and conventional approaches applied in studying the development of *Cordyceps sinensis*.

Stages	Caterpillar-shaped sclerotium formation		Stiff worm	Stroma sprout
Duration for different stages^40^	Around 2 months	About 3–5 days	3–4 months	Around 2 months
Stable carbon isotope analysis (δ^13^C values of subsamples*)	H1 to H2: highest value, initial infection	A3 to A*i*: slightly declined	H1 to S1: sharply jumped down	S1-S*i*: continuously decreased
	H2 to T3: slightly declined, inception			
	T3 to A3: sharply decreased, incubation			
Field observation in morphology	Could not be observed due to no symptom)^40^	The larva started to behave abnormally, and its skin colour gradually changed^31,40^.	Relatively long dormant period^40,43^ (“Winter-worm”)	The stroma started to germinate at the head for more than 2 months and eventually formed the mature stroma^31,41,43^ (“Summer grass”).
Macroscopic observation in the growth of mycelia	The inside became hollow and the integument became moist. Then, a white hyphal coil firstly developed at the pharynx and gradually extended to the whole body^42^.		The stiff worm was gradually coated by mycelia^31^.	1^st^: The stroma kept growth for one month to reach the length of around 3 cm. Then, its apex swelled and was covered with the granulated perithecium.
				2^nd^: The stroma continued to grow for 20 days to reach the final length of about 4.5 cm.
				3^rd^: In the coming 10 days, the stroma would undergo the development period of ascospores, including growth, maturation, and eruption^31,41^.
Microscopic observation in the growth of mycelia	1^st^: The infectious fungus firstly invaded the host and formed several spheroid hyphal bodies.			The inner of a stroma was made up of interwoven mycelia, and finally multiseptate and elongate fusoid ascospores were produced^31^.
	2nd: The hyphal bodies multiplied in the host and gradually formed multinucleate hyphal bodies.			
	3rd: The multinucleate hyphal bodies further developed into mycelia through the following processes: budding multiplication, conglobation and connection, and hyphal body fusion. The mycelia continued to grow and completely filled the host body cavity^15,16,31,41^.			

*S1 to S*i*, H1 to H2, T1 to T3, and A1 to A*i* are the subsamples from the stroma, head, thorax, and abdomen according to their positions, respectively. The italic lower case letter *i* represents the section numbers of the stroma and abdomen, respectively.

There are two naturally occurring stable carbon isotopes, i.e., ^12^C (98.9%) and ^13^C (1.1%). The isotopes are unevenly distributed among and within different compounds, and their distribution can reveal the information on physical, chemical, and metabolic processes involved in carbon transformations^32^. For all the organisms, the metabolism of carbonaceous compounds, whatever catabolism or synthetic metabolism, may cause stable carbon isotope fractionation. Among all the biometabolisms, the fixation and release of CO_2_ are proved to be the most notable factor that can induce stable carbon isotope fractionation. Thus, the stable carbon isotope composition of an autotrophic organism is linked to its photosynthetic fixation of atmospheric CO_2_. It was well recognized that C3 and C4 plants possess the distinctly different δ^13^C values^32^, i.e., −22‰ to −35‰ for C3 plants, while −9‰ to −17‰ for C4 plants, due to the isotope fractionation in the photosynthetic carbon fixation. For a heterotrophic organism, it utilizes foods or culture media^33^ as its carbon source and energy supply. Therefore, the carbon isotope composition depends on its foods or culture media^33^, and CO_2_ is released to the environments via respiration. Thus,^12^C is preferentially consumed in respiration, resulting in the ^13^C enrichment in residual matters or culture media. Accordingly, stable carbon isotope fractionation is closely related to the rate and duration of metabolism. Hence, this difference of stable carbon isotopes may reach a detectable level with the extension of time. Based on the above principle of isotope fractionation, we may study the metabolism and development of organisms as well as the authentication of biological products^6,24–26,34–36^.

**Figure 4:**
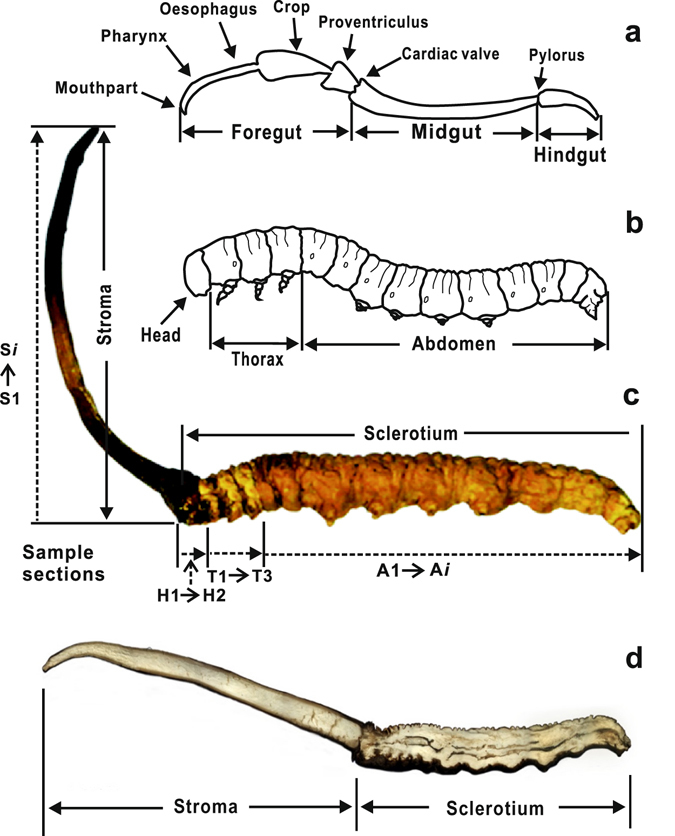
Schematic diagram illustrating the digestive tract (**a**), host larva (**b**), *Cordyceps sinensis* (**c**), and the inner structure of *Cordyceps sinensis* (**d**). The diagram was created using CorelDraw X4 (Corel Corporation, Ontario, Canada).

It can be seen from the cross section along the central line of fresh *C*. *sinensis* (Fig. 4d) that the hemocoelom of the host larva was imbued with white mycelia, and only black food residuals occurred in its deformed digestion guts. Although the distribution of mycelia was apparently homogeneous in the caterpillar-shaped sclerotium, the δ^13^C values were evidently changeable in different parts of the caterpillar-shaped sclerotium, which spatially represented the previous host larva. Their variation exhibits a step-shaped downtrend from the head to thorax, and to the abdomen end, while the δ^13^C values of the head and thorax always maintain in a higher plateau (Fig. 2). This higher step exhibits a slight incline from the head (H1 and H2) to the second segment of thorax (T2). The δ^13^C values sharply decrease from the third segment of thorax to near the third abdominal segment (T3 to A3). Many entomologists have investigated the anatomical structure of the digestive system of a larva, and the results revealed that the digestive tract of one healthy larva might be divided into foregut, midgut and hindgut by cardiac valve and pylorus^37^ (Fig. 4a). The foregut, which extends from the mouth to the cardiac valve, contains preoral cavity (H1), pharynx (H2), oesophagus (T1 and T2), crop, and proventriculus (T3 to A3), and their boundaries are unclear^37^. The pattern of δ^13^C values in the caterpillar-shaped sclerotium well matches the configuration of the digestive tract of the host larva. The maximum δ^13^C values occur in the segment from the mouthpart to the end of oesophagus, in which many tiny tracts are included. The δ^13^C values sharply decrease from the crop to proventriculus, which is suitable for food storage and remastication due to the relatively larger volume^37^ (Fig. 4a). Thus, we infer that the fungus growth may closely be related to the digestive tract of the host larva.

As we know, a mature *T*. larva (approximately fourth or fifth instar) is more easily infected by *O*. *sinensis* from the habitat soils in summer^7,15^. After the hemocoelom of a host larva is attacked by *O*. *sinensis*, its mycelia will grow in the larva at a certain pace, and eventually a fungus-larva symbiote will be formed^15^ (Table 1). Obviously, the δ^13^C variation in the fungus-larva symbiote is mainly attributed to the formation time and activities of the predecessor (host larva) and successor (fungus). Although no report on the metabolic activity of the host *T*. larva is presented so far, the referential physiologic studies on a mature Lepidoptera larva have already been available, i.e., its silk gland, fat body and midgut are three tissues with the highest metabolic activity in the body-cavity^38^. Because the silk gland and fat body are mutually distributed the whole body-cavity of the larva^39^, the metabolic activity in the corresponding positions, including its midgut, may be highest in the larva. Accordingly, the δ^13^C values of its third to sixth abdominal segments should be highest. However, our newly-obtained δ^13^C values exhibit minima in the third to sixth abdominal segments (from A3 to A12) of the fungus-larva symbiote. Comparatively, after the fungus intrudes and colonizes in the host larva for several months, a white hyphal coil will form at the pharynx, and gradually extends to the whole body (Table 1). During this long-term process, *O*. *sinensis*, a heterotrophic fungus, utilizes the host larva as its substrate for keeping tip growth to the whole body. As the extension of time,^13^C will accumulate in the host larva due to the fungus respiration^25^; and the detectable difference of the δ^13^C values between the earlier and later parts colonized by *O*. *sinensis* occurs in the final sclerotium. It has been demonstrated from Table 1 that the δ^13^C variation in the sclerotium is exactly coincident with the developing stages of the colonization. This coincidence suggests that the fungal development may be a more important factor to cause the δ^13^C difference within the sclerotium. It is shown from Fig. 2 that the δ^13^C variation in different parts of the sclerotium exhibits the same downward trend with the highest value at the head (H1 and H2) and the first to second segment (T1 and T2) of the thorax. Owing to the existence of a pair of hard and smooth capsules in the larva head, the head has the ability to prevent various pathogen infection^39^. Therefore, we consider that the part in the sclerotium of *C*. *sinensis* with the highest δ^13^C value may firstly be formed, and the site near the boundary (ecdysial line) between the head and thorax in the host larva may initially be attacked by *O*. *sinensis*. Our inference is consistent with the previous result^14^ and also supports the hypothesis of digestive system infection.

Based on the observations on the development of *O*. *sinensis* mycelia by microscope (Table 1), the formers put forward the hypothesis of digestive system infection^15^. They considered that the infectious fungus firstly invaded the oral route with the food into the digestive tract, and then the mycelia intruded into the blood chamber through the digestive tract wall. The occurrence of a great amount of hyphae in the blood cavity observed under a microscope supports this hypothesis^16^. With the growth and development of mycelia, the caterpillar-shaped hard sclerotium (cadaver) is gradually developed by the knotted mycelia. Finally, *C*. *sinensis* is formed once the stroma germinates. The above-described whole process lasts more than one and a half years^16^ (Table 1). Based on the newly-obtained δ^13^C values of *C*. *sinensis*, we may further clarify the stages of infection and development as below.

According to previous observations^40^, after the fungus initially invaded into the host larva, its mycelia underwent a growth period of 80 to 100 days. During this period, both the appearance and behaviors of the infected larva were normal (Table 1). Our obtained data proved the existence of this symptom-free stage and provided the information on the following developmental processes.

The host larva is an omnivorous insect, which leads its troglodytic life at the depth of 5–25 cm in soils, and prefers to feed on the tender plant roots or humic substances^6^. The larva may be infected by *O*. *sinensis* from habitat soils through a feeding process. Thus, the mouthpart of the host larva is a more preferred initial target invaded by the infectious fungus. The δ^13^C values from the mouthpart to the end of oesophagus (H1 to T2) are evidently higher than those of the other subsamples. Thus, we consider that the mycelia gradually grew and accordingly extended to the end of oesophagus after the fungus firstly attacked the mouthpart of the host larva. The narrow variation in the δ^13^C values suggests that this process is completed relatively quickly.

The δ^13^C values sharply jump down from T3 to A3, implying that the mycelia extended very slowly in the crop and proventriculus. In Fig. 3, the decreasing range from T3 to A3 varies with each sample of *C*. *sinensis*, which may be caused by different rates and durations of the mycelia growth in individual sample. Therefore, we consider that there is a competitive relation between the immune system of a host larva and the fungus at the early stage, and the growth rate of the fungus in the host larva depends on its status in the competition^7,41^. For instance, if the immune system of the host larva has been strong enough to resist the fungus invasion, the fungus would grow slowly, resulting in the larger decreasing amplitude in the δ^13^C values.

Our above speculation is consistent with the previous observations^42^. After dissecting numerous host larvae, which were alive but already infected by the fungus, Qi found that the morphological features inside the host larva abnormally changed (Table 1). Firstly, the inside became hollow and the integument became moist. Then, a white hyphal coil firstly developed at the pharynx and gradually extended to the whole body^42^. However, she did not provide more detailed experimental data on the early development of mycelia, due to the limitation of previous methods. Combined with the newly-obtained δ^13^C data, we may speculate that, after just undergoing the development stage, the fungus may go through an incubation period, either long or short as revealed in different *C*. *sinensis* samples. Finally, an initial hyphal coil crawls through the foregut.

According to the previous observations^40^, after the asymptomatic stage of 80–100 days, the larva starts to behave abnormally, i.e., firstly crawling more frequently, then becoming dully, and finally converting into a hard muscardine cadaver (Table 1). This dominant period may last only 3 to 5 days. The caterpillar-shaped sclerotium does not sprout a stroma in winter, usually till in late spring after nearly 4 months^43^. Coincidently, our novel data provide powerful evidence for the occurrence of this stage.

The δ^13^C values from A3 to the end of abdomen maintain a stable and low level (Fig. 2), showing that the fungus begins to grow fast and rapidly extend to the end of abdomen, and eventually the hard caterpillar-shaped sclerotium is formed. Because this process may last a short period (3–5 days)^16,40^, there is no evident carbon isotope fractionation (Fig. 2). Thus, we consider that the rapid growth of the fungus in this period may result in the abnormal behaviors of the host larva.

The δ^13^C values sharply jump down from the head of caterpillar-shaped sclerotium (H1) to the bottom of stroma (S1) (Fig. 2). This abrupt decrease of the δ^13^C values and the maintenance of lower δ^13^C values from caterpillar-shaped sclerotium (H1) to the bottom of stroma (S1) suggest that the fungus may undergo a relatively long dormant period (generally 3–4 months) between the formation of caterpillar-shaped sclerotium and the sprout of stroma^40^.

The δ^13^C values continuously decreased from the bottom to the top of stroma, and the decreasing interval varies with each sample of *C*. *sinensis* (Fig. 3). This result indicates that since sprouting from the head of the caterpillar-shaped sclerotium, each stroma has remained its tip growth with different growth rates, as shown by the inconsistent decrease amplitude of the δ^13^C values of each stroma (Fig. 3). Previous studies demonstrate that the stroma usually starts to germinate at the head of the caterpillar-shaped sclerotium in late spring under the appropriate conditions^31,41^ and then may keep tip growth for around 2.5 months. The average growth rate was approximately 0.55 mm/d, and the average length of mature stromata eventually formed was 41.1 ± 5.3 mm^31^ (Table 1). An evidently negative correlation of the δ^13^C values among different positions of each stroma in five samples is revealed in this study, implying that the variation trend of their δ^13^C values may indicate the stroma growth.

In summary, the nearly similar δ^13^C profiles were revealed for the samples of *C*. *sinensis* from five representative high-altitude habitats by using stable carbon isotope analysis. The δ^13^C profiles are characterized by the occurrence of the δ^13^C maximum at the head, a slight decrease from the head to the end of thorax, and a sharp descent trend from the end of thorax to the forepart of abdomen, which indicate that the site near the head of the host larva may be the target initially attacked and colonized by *O*. *sinensis*. Our newly-obtained data prefer to support the hypothesis of digestive system infection in the host larva.

For more than one thousand years, *C*. *sinensis* has been glorified for the predominant therapeutic effect and esteemed as a valuable herbal medicine for its mysterious life history in the Qinghai-Tibetan Plateau. Meanwhile, this mystery also induces over excavation, which has caused serious environmental and ecological issues in the local region. In this paper, stable carbon isotope analysis has been employed to reveal a mysterious legend on the fungus-larva relation, but the spatial heterogeneity of the δ^13^C values in uninfected larvae needs to be revealed via one specific experiment in the further study. Overall, it is prospected that this idea may be applied in tracing this similar linkage between microbe and insect in the follow-up case studies.

## Materials and Methods

### Sampling

Five representative samples of *C*. *sinensis* were collected from the habitats in Sichuan, Qinghai and Tibet in June 2014. The sampling sites are illustrated in Fig. 1. The lengths of the stroma and the host caterpillar of *C*. *sinensis* are 3.3 cm and 4.0 cm for sample a at Changdu, 3.6 cm and 4.2 cm for sample b at Guoluo, 3.5 cm and 3.5 cm for sample c at Haxiu, 3.0 cm and 3.8 cm for sample d at Litang, and 3.5 cm and 3.7 cm for sample e at Naqu, respectively (Fig. 1). Briefly, the samples of *C*. *sinensis* after vacuum freeze-drying were carefully cloven nearly along its central line with a scalpel (Fig. 4d). The food residues in previous digestive tracts of the host caterpillar were carefully removed with a needle. After that, the subsamples at different positions in one clove were accordingly prepared. Each sample of *C*. *sinensis* was sectioned into approximately 40 pieces from the stroma top to the host caterpillar end, and the thickness of each piece is approximately 2 mm. These sections were divided into four groups on the basis of their positions (Fig. 4c), i.e., stroma, S1 to S*i* (the italic lower case letter *i* represents the number of the total sections of the stroma); head, H1 to H2 (the subsamples were sectioned from the head of caterpillar-shaped sclerotium); thorax, T1 to T3 (the subsamples were sectioned from the three thorax segments of the caterpillar-shaped sclerotium in sequence); and abdomen, A1 to A*i* (the italic lower case letter *i* means the number of the total sections of the abdomen). More details of the subsamples are presented in Table S1 and Fig. 4c. The subsamples at different positions in the other clove were performed the same operation as the parallel samples.

### Analysis of stable carbon isotope ratios (δ^13^C) of *C*. *sinensis*

The stable carbon isotope ratios (δ^13^C) of *C*. *sinensis* were determined by elemental analyser-isotope ratio mass spectrometer (EA-IRMS) at the Laboratory of Bioorganic Geochemistry, School of Marine Sciences, Sun Yat-Sen University, Guangzhou, China. About 0.5–1.0 mg of each subsample with the grain size of less than 100 meshes was weighed and loaded into a clean tin capsule. The capsules containing subsamples were placed on an automatic feeder for solid samples equipped for the vario ISOTOPE cube elemental analyser (Elementar, Hanau, Germany), and were burned in an ultrapure O_2_ atmosphere in the CuO combustion tube with its temperature set at 950 °C. Combustion gases were eluted through a reduction column by a stream of He gas and passed into the gas chromatograph where CO_2_, still in the He stream, was separated from the other gases. The gas stream then entered an IsoPrime 100 isotope ratio mass spectrometer (Elementar, Manchester, UK) where the CO_2_ gas was analysed by comparison with the reference CO_2_ gas with a known δ^13^C value (−33.24‰, calibrated against the IAEA-NBS-22 reference material with a δ^13^C value of −30.03 ± 0.04‰). During every batch of analyses, an empty tin capsule was analysed as the blank to check the background, and a reference material sulfanilamide with a known δ^13^C value (−28.13 ± 0.02‰) was used to check the reproducibility and accuracy. Low background (the peak height was less than 0.01 V), which was much lower than the peak height of the sample (greater than 1.5 V), and excellent reproducibility and accuracy were achieved. The corresponding standard deviations of the analysis and the deviations between the measured data and predetermined data were ± 0.15‰. The stable carbon isotope ratio of *C*. *sinensis* was expressed by the conventional delta (δ) notation:

1 $${{\rm{\delta }}}^{13}{\rm{C}}(\textperthousand )=[({R}_{{\rm{sample}}}/{R}_{{\rm{std}}})\,-\,1]\times {10}^{3}$$

where *R*_sample_ and *R*_std_ are the ^13^C/^12^C isotope ratios corresponding to each subsample and V-PDB standard, respectively^44,45^.

### Statistical analysis

The experimental data of the subsamples were analysed using the IBM SPSS Statistics (Ver. 20, Microsoft, USA). The δ^13^C data of each subsample was the average value of thrice determinations with a relative standard deviation (RSD) of less than 0.5%. The SD for each pair of the parallel samples was less than 0.2‰. The correlation of the variation trends in the δ^13^C values among five samples of *C*. *sinensis* were analysed by Spearman’s rank correlation analysis (*p* < 0.05), exhibiting a significant correlation. The correlation between the Δ^13^C value and the position (*i*) of each stroma was analysed using Pearson’s linear correlation analysis (*p* < 0.05), exhibiting a significantly negative correlation at the 0.01 level. Their correlation equations are expressed as below: Δ^13^C = −0.077 *i* − 0.741 for sample a (*r*² = 0.951), Δ^13^C = −0.040 *i *− 0.191 for sample b (*r*² = 0.970), Δ^13^C = −0.063 *i* − 1.223 for sample c (*r*² = 0.841), Δ^13^C = −0.043 *i* − 0.996 for sample d (*r*² = 0.811), and Δ^13^C = −0.103 *i* − 0.938 for sample e (*r*² = 0.987).

### Data Availability

All data generated or analysed during this study are included in this published article (and its Supplementary Information files).

## Electronic supplementary material

Supplementary Table S1
