# Publisher Correction: Fungus-larva relation in the formation of *Cordyceps sinensis* as revealed by stable carbon isotope analysis

**DOI:** 10.1038/s41598-018-23242-4

**Published:** 2018-03-19

**Authors:** Lian-Xian Guo, Yue-Hui Hong, Qian-Zhi Zhou, Qing Zhu, Xiao-Ming Xu, Jiang-Hai Wang

**Affiliations:** 1 Dongguan Key Laboratory of Environmental Medicine, School of Public Health, Guangdong Medical University, Dongguan, Guangdong, 523808 People’s Republic of China; 2 Guangdong Provincial Key Laboratory of Marine Resources and Coastal Engineering/South China Sea Bioresource Exploitation and Utilization Collaborative Innovation Center, School of Marine Sciences, Sun Yat-Sen University, Guangzhou, 510006 People’s Republic of China

Correction to: *Scientific Reports* 10.1038/s41598-017-08198-1, published online 10 August 2017

This Article contains an error in the formatting of Table 1. The correct Table 1 appears below.

**Table 1:** Comparison between stable carbon isotope analysis and conventional approaches applied in studying the development of *Cordyceps sinensis*.

Stages	Caterpillar-shaped sclerotium formation		Stiff worm	Stroma sprout
Duration for different stages^40^	Around 2 months	About 3–5 days	3–4 months	Around 2 months
Stable carbon isotope analysis (δ^13^C values of subsamples*)	H1 to H2: highest value, initial infection	A3 to A*i*: slightly declined	H1 to S1: sharply jumped down	S1-S*i*: continuously decreased
	H2 to T3: slightly declined, inception			
	T3 to A3: sharply decreased, incubation			
Field observation in morphology	Could not be observed due to no symptom)^40^	The larva started to behave abnormally, and its skin colour gradually changed^31,40^.	Relatively long dormant period^40,43^ (“Winter-worm”)	The stroma started to germinate at the head for more than 2 months and eventually formed the mature stroma^31,41,43^ (“Summer grass”).
Macroscopic observation in the growth of mycelia	The inside became hollow and the integument became moist. Then, a white hyphal coil firstly developed at the pharynx and gradually extended to the whole body^42^.		The stiff worm was gradually coated by mycelia^31^.	1^st^: The stroma kept growth for one month to reach the length of around 3 cm. Then, its apex swelled and covered with the granulated perithecium.
				2^nd^: The stroma continued to grow for 20 days to reach the final length of about 4.5 cm.
				3^rd^: In the coming 10 days, the stroma would underwent the development period of ascospores, including growth, maturation, and eruption^31,41^.
Microscopic observation in the growth of mycelia	1^st^: The infectious fungus firstly invaded the host and formed several spheroid hyphal bodies.			The inner of a stroma was made up of interwoven mycelia, and finally multiseptate and elongate fusoid ascospores were produced^31^.
	2^nd^: The hyphal bodies multiplied in the host and gradually formed multinucleate hyphal bodies.			
	3^rd^: The multinucleate hyphal bodies further developed into mycelia through the following processes: budding multiplication, conglobation and connection, and hyphal body fusion. The mycelia continued to grow and completely filled the host body cavity^15,16,31,41^.			

^*^S1 to S*i*, H1 to H2, T1 to T3, and A1 to A*i* are the subsamples from the stroma, head, thorax, and abdomen according to their positions, respectively. The italic lower case letter *i* represents the section numbers of the stroma and abdomen, respectively.

