# Supplementary material for: Fungus-larva relation in the formation of Cordyceps sinensis as revealed by stable carbon isotope analysis
Source: Sci Rep. 2017 Aug 10;7:7789. doi: 10.1038/s41598-017-08198-1 (PMC5552863; doi:10.1038/s41598-017-08198-1)
Supplement: Supplementary file 1 — Supplementary Table S1 [file 41598_2017_8198_MOESM1_ESM.pdf]

Supplementary Information

Journal name: Scientific Reports

Manuscript title: Fungus-larva relation in the formation of *Cordyceps sinensis* as revealed by stable carbon isotope analysis

Authors: Lian-Xian Guo[1,2], Yue-Hui Hong[2], Qian-Zhi Zhou[2], Qing Zhu[2], Xiao-Ming Xu[2*] & Jiang-Hai Wang[2*]

[1] Dongguan Key Laboratory of Environmental Medicine, School of Public Health, Guangdong Medical University, Dongguan, Guangdong 523808, People's Republic of China

[2] Guangdong Provincial Key Laboratory of Marine Resources and Coastal Engineering/South China Sea Bioresource Exploitation and Utilization Collaborative Innovation Center, School of Marine Sciences, Sun Yat-Sen University, Guangzhou 510006, People's Republic of China

[*]Authors to whom correspondence should be addressed. E-mails: wangjhai@mail.sysu.edu.cn (J.-H. Wang); xxm8302@126.com (X.-M. Xu). Tel.: +86 20 39332212; Fax: +86 20 85261499.

Captions: this file contains a supplementary table (Table S1).

Table S1. Stable carbon isotope composition of *C. sinensis* from five representative habitats in the Qinghai-Tibetan Plateau and its adjacent high-altitude areas.

Sample a			Sample b			Sample c			Sample d			Sample e		
Subsample	$\delta^{13}C$ (‰)	$\Delta^{13}C$ (‰)	Subsample	$\delta^{13}C$ (‰)	$\Delta^{13}C$ (‰)	Subsample	$\delta^{13}C$ (‰)	$\Delta^{13}C$ (‰)	Subsample	$\delta^{13}C$ (‰)	$\Delta^{13}C$ (‰)	Subsample	$\delta^{13}C$ (‰)	$\Delta^{13}C$ (‰)
S17	-27.87	-2.19	S21	-27.91	-1.10	S17	-28.60	-2.20	S16	-27.99	-1.66	S15	-28.35	-2.45
S16	-27.69	-2.01	S20	-27.83	-1.02	S16	-28.46	-2.06	S15	-27.97	-1.64	S14	-28.28	-2.38
S15	-27.66	-1.98	S19	-27.80	-0.99	S15	-28.58	-2.18	S14	-27.96	-1.63	S13	-28.23	-2.33
S14	-27.44	-1.76	S18	-27.76	-0.95	S14	-28.42	-2.02	S13	-27.95	-1.62	S12	-28.05	-2.15
S13	-27.37	-1.69	S17	-27.67	-0.86	S13	-28.59	-2.19	S12	-27.90	-1.57	S11	-27.94	-2.04
S12	-27.34	-1.66	S16	-27.57	-0.76	S12	-28.30	-1.90	S11	-27.70	-1.37	S10	-27.93	-2.03
S11	-27.27	-1.59	S15	-27.56	-0.75	S11	-28.47	-2.07	S10	-27.68	-1.35	S9	-27.81	-1.91
S10	-27.13	-1.45	S14	-27.51	-0.70	S10	-28.25	-1.85	S9	-27.73	-1.40	S8	-27.74	-1.84
S9	-26.98	-1.30	S13	-27.45	-0.64	S9	-28.44	-2.04	S8	-27.60	-1.27	S7	-27.53	-1.63
S8	-26.93	-1.25	S12	-27.49	-0.68	S8	-28.19	-1.79	S7	-27.64	-1.31	S6	-27.35	-1.45
S7	-27.01	-1.33	S11	-27.47	-0.66	S7	-28.32	-1.92	S6	-27.57	-1.24	S5	-27.35	-1.45
S6	-26.78	-1.10	S10	-27.43	-0.62	S6	-27.88	-1.48	S5	-27.66	-1.33	S4	-27.22	-1.32
S5	-26.75	-1.07	S9	-27.37	-0.56	S5	-27.98	-1.58	S4	-27.63	-1.30	S3	-27.24	-1.34
S4	-26.84	-1.16	S8	-27.38	-0.57	S4	-27.76	-1.36	S3	-27.66	-1.33	S2	-27.04	-1.14
S3	-26.80	-1.12	S7	-27.36	-0.55	S3	-27.81	-1.41	S2	-27.25	-0.92	S1	-26.92	-1.02
S2	-26.65	-0.97	S6	-27.21	-0.40	S2	-27.53	-1.13	S1	-27.23	-0.90	H1	-25.90	0.00
S1	-26.47	-0.79	S5	-27.18	-0.37	S1	-27.69	-1.29	H1	-26.33	0.00	H2	-25.99	-0.09
H1	-25.68	0.00	S4	-27.15	-0.34	H1	-26.40	0.00	H2	-26.35	-0.02	T1	-25.97	-0.07
H2	-25.72	-0.04	S3	-27.13	-0.32	H2	-26.51	-0.11	T1	-26.33	0.00	T2	-26.24	-0.34
T1	-25.71	-0.03	S2	-27.05	-0.24	T1	-26.61	-0.21	T2	-26.39	-0.06	T3	-26.27	-0.37
T2	-25.78	-0.10	S1	-27.08	-0.27	T2	-26.64	-0.24	T3	-26.38	-0.05	A1	-26.39	-0.49

T3	-25.93	-0.25	H1	-26.81	0.00	T3	-26.79	-0.39	A1	-26.43	-0.10	A2	-26.49	-0.59
A1	-26.23	-0.55	H2	-26.83	-0.02	A1	-27.58	-1.18	A2	-26.94	-0.61	A3	-27.67	-1.77
A2	-26.63	-0.95	T1	-26.83	-0.02	A2	-27.65	-1.25	A3	-27.36	-1.03	A4	-27.43	-1.53
A3	-26.81	-1.13	T2	-26.84	-0.03	A3	-27.48	-1.08	A4	-27.48	-1.15	A5	-27.58	-1.68
A4	-27.13	-1.45	T3	-26.94	-0.13	A4	-27.69	-1.29	A5	-27.36	-1.03	A6	-27.63	-1.73
A5	-27.18	-1.50	A1	-27.64	-0.83	A5	-27.63	-1.23	A6	-27.44	-1.11	A7	-27.35	-1.45
A6	-27.09	-1.41	A2	-28.47	-1.66	A6	-27.41	-1.01	A7	-27.33	-1.00	A8	-27.53	-1.63
A7	-27.30	-1.62	A3	-28.35	-1.54	A7	-27.50	-1.10	A8	-27.54	-1.21	A9	-27.43	-1.53
A8	-26.99	-1.31	A4	-28.52	-1.71	A8	-27.55	-1.15	A9	-27.37	-1.04	A10	-27.33	-1.43
A9	-27.25	-1.57	A5	-28.69	-1.88	A9	-27.45	-1.05	A10	-27.21	-0.88	A11	-27.59	-1.69
A10	-26.95	-1.27	A6	-28.43	-1.62	A10	-27.56	-1.16	A11	-27.27	-0.94	A12	-27.49	-1.59
A11	-27.22	-1.54	A7	-28.46	-1.65	A11	-27.51	-1.11	A12	-27.44	-1.11	A13	-27.55	-1.65
A12	-26.87	-1.19	A8	-28.72	-1.91	A12	-27.64	-1.24	A13	-27.16	-0.83	A14	-27.35	-1.45
A13	-26.98	-1.30	A9	-28.67	-1.86	A13	-27.47	-1.07	A14	-27.37	-1.04			
A14	-26.91	-1.23	A10	-28.53	-1.72				A15	-27.21	-0.88			
A15	-26.79	-1.11	A11	-28.75	-1.94				A16	-27.24	-0.91			
A16	-26.84	-1.16	A12	-28.75	-1.94									
			A13	-28.64	-1.83									
			A14	-28.58	-1.77									
			A15	-28.53	-1.72									

Each sample of *C. sinensis* was sectioned into approximately 40 subsamples from the stroma top to the sclerotium end. S1 to S*i*, H1 to H2, T1 to T3, and A1 to A*i* are the subsamples from the stroma, head, thorax, and abdomen according to their positions, respectively. All the $\delta^{13}C$ values are the means of thrice determinations, and have the relative standard deviations less than 0.50%; and the $\Delta^{13}C$ values represent the differences of the $\delta^{13}C$ values between the head (H1) and other parts in each sample of *C. sinensis*.
